# Exploring the effect of the structural model of active aging on the self-assessment of quality of life among older people: A cross-sectional and analytical study

**DOI:** 10.1590/1516-3180.2022.0609.R1.230523

**Published:** 2024-03-11

**Authors:** Nayara Gomes Nunes Oliveira, Alisson Fernandes Bolina, Vanderlei José Haas, Darlene Mara dos Santos Tavares

**Affiliations:** I PhD. Nurse, specialist in older people health, Hospital de Clínicas, Universidade Federal de Uberlândia (UFU), Uberlândia (MG), Brazil.; II PhD. Nurse and Adjunct Professor, Universidade de Brasília (UnB), Brasília (DF), Brazil.; III PhD. Physicist and Professor, Postgraduate Program in Health Care, Universidade Federal do Triângulo Mineiro (UFTM), Uberaba (MG), Brazil.; IV PhD. Nurse and Associate Professor, Department of Nursing Education and Community Health, Nursing Graduate Program, Universidade Federal do Triângulo Mineiro (UFTM), Uberaba (MG), Brazil.

**Keywords:** Aged, Quality of life, Models, statistical, Older adults, Quality of life of older people, Active aging, Statistical model

## Abstract

**BACKGROUND:**

Although studies have examined the relationship between variables associated with active aging and quality of life (QoL), no studies have been identified to have investigated the effect of a structural model of active aging on QoL in a representative sample of older people in the community.

**OBJECTIVE:**

To measure the domains and facets of QoL in older people and identify the effect of the structural model of active aging on the self-assessment of QoL.

**DESIGN AND SETTING:**

This cross-sectional analytical study included 957 older people living in urban areas. Data were collected from households using validated instruments between March and June 2018. Descriptive, confirmatory factor, and structural equation modeling analyses were performed.

**RESULTS:**

Most older people self-rated their QoL as good (58.7%), and the highest mean scores were for the social relationships domain (70.12 ± 15.4) and the death and dying facet (75.43 ± 26.7). In contrast, the lowest mean scores were for the physical domains (64.41 ± 17.1) and social participation (67.20 ± 16.2) facets. It was found that active aging explained 50% of the variation in self-assessed QoL and directly and positively affected this outcome (λ = 0.70; P < 0.001).

**CONCLUSION:**

Active aging had a direct and positive effect on the self-assessment of QoL, indicating that the more individuals actively aged, the better the self-assessment of QoL.

## INTRODUCTION

Active aging is defined as “the process of optimizing opportunities for health, participation, and safety, to improve the quality of life (QoL) as people age.”^1^ In the theoretical model proposed by the World Health Organization (WHO), this paradigm contemplates the determinants of culture and gender, which are considered transversal factors that shape people and the environment in which they live throughout life; behavioral and personal, which are specific to each individual; and physical environment, social, economic, social services, and health, which constitute the contextual factors.^1^

In this scenario, the promotion of active aging has its primary objective to maintain or improve QoL.^1^ It can be understood as the “individuals’ perception of their position in life concerning the context and value systems in which they fit in, including its objectives, expectations, standards, and concerns.”^2^ Thus, its complexity is highlighted in the face of the interaction of components such as physical and psychological health, level of functional independence and social relationships, and the environment.^3^

Among older people, QoL can be assumed to be very good, or at least preserved, when they remain active, independent, and in good physical health and social relationships.^4,5^ Although studies have investigated the relationship between variables associated with active aging and QoL,^6-9^ no identified studies have verified the effect of a structural model of active aging according to the multidimensional design proposed by the WHO^1^ on QoL in a representative sample of older adults in a community.

Notably, structural equation modeling (SEM) makes it possible to understand the interrelationship between multiple variables,^10^ which enables the operationalization of the expanded concept of active aging adopted in this study,^1^ and to analyze its effect on the QoL of older people. The findings of this study may raise reflections on advanced gerontological practices in line with the current models of healthcare provision (which are not strictly focused on the physical aspects of senescence and senility), but, above all, on a multidimensional approach to active aging.

## OBJECTIVE

This study aimed to measure the domains and facets of the QoL of older people in the community and identify the effect of the structural model of active aging on the self-assessment of QoL.

## METHOD

### Design

The Strengthening the Reporting of Observational Studies in Epidemiology tool guided this cross-sectional and analytical study. Further, the study employed a quantitative approach and was a part of a larger project titled “Active Aging, Global Functionality and Quality of Life among Older People in the Uberaba Health Microregion (Minas Gerais),” developed in the urban area of a health microregion in the state of Minas Gerais. This microregion consists of eight municipalities and comprises 57% of the older population of the Southern Triangle Macroregion.

### Sample/Participants

The sample size calculation was based on the prevalence rate of 28.8% for lower participation in instrumental activities of daily living,^11^ aiming for an accuracy of 3.0%, and a 95% confidence interval for a finite population of 43,166. Consequently, a minimum sample size of 858 older people was achieved. Considering a sample loss of 20%, the maximum number of attempts made was 980 older people.

Multistage cluster sampling was used for population selection. The first stage considered the arbitrary drawing using systematic sampling of 50% of the census tracts in each municipality in a health microregion. For each municipality, the number of households selected was calculated proportionately to the number of older adults residing in the eight cities in that region. The number of households was then divided by the number of census tracts to obtain a similar number of older people to be interviewed in each census tract. Finally, the first household was randomly selected in each census sector, and the others were selected in a standardized sense until the sector sample was saturated. Notably, one older person was recruited per household; if one more person aged 60 years or older was residing in that place, the person who had first contact with the interviewer was interviewed.

The inclusion criteria included individuals aged 60 years or older living in an urban area of a health microregion in Minas Gerais. Institutionalized older people were excluded if they had communication problems, such as deafness not corrected by devices, severe speech disorders, cognitive decline according to the Mini-Mental State Examination (MMSE),^12^ no informant to answer the Functional Activities Questionnaire (FAQ),^13^ or a final score greater than or equal to six points in the FAQ.

Based on the eligibility criteria, 977 older people were interviewed; in this sample, 15 had severe cognitive decline, and five did not undergo a full interview. Therefore, 957 older adults were included in this study.

### Data collection

Interviews were conducted at the homes of older people from March to June 2018. Trained interviewers with previous experience in collecting data conducted these interviews. Five previously selected supervisors checked the interviews to verify the completion and consistency of the items and ensure quality control.

Cognitive decline was assessed using the MMSE, considering the following cutoff points: ≤ 13 for illiterate, ≤ 18 for low (1–4 incomplete years) and medium (4–8 incomplete years) education, and ≤ 26 for high (≥ 8 full years) education.^12^ If an older person presented a cognitive decline in the MMSE assessment, the informant was asked to participate, and the FAQ was applied, which verifies the presence and severity of cognitive decline based on the assessment of functionality and the need for assistance from other individuals.^13^ The FAQ associated with the MMSE indicates the most severe presence of cognitive decline when the score is greater than or equal to 6 points.^13^

Sociodemographic and economic data were obtained through a structured questionnaire, which was elaborated upon and widely used by Collective Health Research Group members.

QoL was assessed based on the application of the World Health Organization Quality of Life-BREF (WHOQOL-BREF), which is composed of four domains: (1) physical, (2) psychological, (3) social relationships, and (4) environment,^3^ and the World Health Organization Quality of Life-OLD (WHOQOL-OLD), which is a specific instrument for the older population, consisting of six facets: (1) functioning of the senses; (2) autonomy; (3) past, present, and future activities; (4) social participation; (5) death and dying; and (6) intimacy,^14^ both validated in Brazil. Notably, the domains and facets of these instruments are composed of questions whose scores on a Likert scale vary according to the degree of satisfaction (1–5 points). The final scores (0–100 points) were calculated using Syntax, with the highest value corresponding to the best QoL.

The self-assessment of QoL was measured using the question, “How would you assess your quality of life?” This question had five response options: very poor, poor, not bad/not good, good, or very good. Notably, the questions regarding QoL were answered based on the last two weeks of life.

Furthermore, a structural model of active aging developed in a previous study, based on the theoretical framework of the WHO,^1^ was used.^15^ The instruments applied at a single moment to measure the determinants of the active aging model were defined considering the most used ones in gerontology and validated in Brazil (Chart 1).^15-29^

**Chart 1:** Instruments used to measure the determinants of the active aging model and categorization of the observed variables

Behavioral determinant		
Measured determinants	Instruments	Code in Structural Equation Modeling
**Anthropometric profile**	Body Mass Index;^16^ Abdominal Circumference;^17^ Calf Circumference;^18^ and Brachial Circumference.^19-20^	Number of suitable items (0 to 4).
**Healthy life habits**		
Alcohol consumption	Do you usually consume alcoholic beverages?	Number of healthy life habits (0 to 4).
Sleep quality	Do you have any trouble sleeping?	
Physical activity	International Physical Activity Questionnaire.^21^	
Smoking	Do you smoke?	
**Self-care practices**		
Attitude towards taking medicines	Instrument for assessing attitude towards medication.^22^	Number of self-care practices (0 to 5).
Vaccination status	Assessment of the vaccination card of elderly individuals.^23^	
Preventive examinations	Have you undergone a preventive examination last year?	
Oral health	When was the last time you went to the dentist?	
Routine consultation	Did you undergo a routine check-up last year?	
**Personal**		
**Measured determinants**	**Instruments**	**Code in Structural Equation Modeling**
**Cognitive ability**	Mini-Mental State Examination.^12^	No (1); Yes (0).
**Resilience**	Connor-Davidson Resilience Scal*e* for Brazil-25.^24-25^	Resilience score.
**Depression, Emotional**	Geriatric Depression Scale.^26^	Number of depressive symptoms.
**Functioning of the senses**	How would you assess the functioning of hearing, vision, taste, smell, and touch?	Very bad (1); Bad (2); Neither bad nor good (3); Good (4), and Very good (5).
**Family history of Chronic Non-Communicable Diseases**	Brazilian Questionnaire of Functional and Multidimensional Assessment.^27^	No (1); Yes (0).
**Morbidities**	Brazilian Questionnaire of Functional and Multidimensional Assessment.^27^	Number of morbidities.
**Physical Environment**		
**Measured determinants**	**Instruments**	**Code in Structural Equation Modeling**
**Physical security and protection**	Do you feel safe in your daily life?	Nothing (1); Very little (2); So-so (3); Quite (4); Extremely (5).
**Physical environment**	Is your physical environment (climate, noise, pollution) healthy?	Nothing (1); Very little (2); So-so (3); Quite (4); Extremely (5).
**Means of transport**	Are you satisfied with your means of transport?	Nothing (1); Very little (2); So-so (3); Quite (4); Extremely (5).
**Environment in the home**	Are you satisfied with the conditions of your place of residence?	Nothing (1); Very little (2); So-so (3); Quite (4); Extremely (5).
**Social**		
**Measured determinants**	**Instruments**	**Code in Structural Equation Modeling**
**Personal relationships**	How satisfied are you with your personal relationships?	Very dissatisfied (1); Dissatisfied (2); Neither dissatisfied nor satisfied (3); Satisfied (4); Very satisfied (5).
**Community activities**	Are you satisfied with the opportunities you have to participate in community activities?	Very dissatisfied (1); Dissatisfied (2); Neither dissatisfied nor satisfied (3); Satisfied (4); Very satisfied (5).
**Social network**	Network and social support scale.^28^	Number of relatives and friends.
**Social support**	Network and social support scale.^28^	Social support score.
**Education years**	How many full years of study do you have?	Full years of study.
**Out-of-school activities**	To what extent do you have opportunities for leisure activities?	Nothing (1); Very little (2); So-so (3); Quite (4); Extremely (5).
**Advanced Activities of daily living**	Thirteen questions of a social nature.^29^	Number of activities performed.
**Economic**		
**Measured determinants**	**Instruments**	**Code in Structural Equation Modeling**
**Paid work**	Do you have paid work?	Yes (1); No (0).
**Monthly individual income**	What is your individual monthly income?	<1 (1); 1┤3 and ≥3.
**Money to meet basic needs**	Do you have enough money to meet your needs?	Nothing (1); Very little (2); So-so (3); Quite (4); Extremely (5).
**Assessment of economic condition**	How do you assess your economic condition?	Good (1); Bad (0).
**Retirement and pension**	Are you a retiree or pensioner?	Yes (1); No (0).
**Social and health services**		
**Measured determinants**	**Instruments**	**Code in Structural Equation Modeling**
**Self-assessment of the course of health status**	Comparing your health today with that of a year ago, would you say your health is worse, equal, or better?^27^	Worse (1); Equal (2); Best (3).
**Assessment of current health status**	How do you assess your health? ^27^	Terrible (1); Bad (2); Regular (3); Good (4); Great (5).
**Access to health care services**	Are you satisfied with your access to health services?	Very dissatisfied (1); Dissatisfied (2); Neither dissatisfied nor satisfied (3); Satisfied (4); Very satisfied (5).
**Link with the health service**	Do you usually seek the same health service when you need care?	Yes (1); No (0).
**Access to continuous-use medicines**	Do you have access to continuous medicines?	Yes (1); No (0).

Fonte: Oliveira et al.^15^

Chart 1 shows the instruments used for data collection and categorization of the observed variables in the SEM analysis.

### Data analysis

An electronic database was built using Excel (Microsoft, Redmond, Washington, United States) with double entries. After checking for inconsistencies between the two databases, the data were imported into the SPSS (IBM, Armonk, New York, United States) version 22.0 and Analysis of Moment Structures (AMOS – SPSS, IBM, Armonk, New York, United States).

The data were subjected to absolute and relative frequency analyses for categorical variables and mean and standard deviation for quantitative variables. A confirmatory factor analysis was performed using AMOS version 23.0 and SPSS version 22.0. This was to identify the effect of active aging on the QoL of older people and assess the quality of fit of the measurement model to the correlational structure among the observed variables.^10^

In the adjustment of the model, the identification strategy of the causal model with latent variables in two steps (two-step) was used: (1) specifying and identifying the measurement sub-model and (2) identifying the structural sub-model, that is, establishing the trajectories for endogenous latent variables.^10^ This method ensures that the measurement model is adequately validated and makes it possible to assess the plausibility of the structural model after ensuring the quality of the measurement model.^10^

In Step 1 of the two-step strategy, a structural model of active aging was used, as described in a previous study.^15^

In both stages, the parameters were estimated using the asymptotic distribution-free method, which is the most traditional method used in SEM analysis.^10^ We also previously conducted an analysis of normality for the items observed through the asymmetry coefficients (sk) and kurtosis (ku), considering the deviation from normality sk indices > 3 and ku > 10.^10^

The quality of the global fit of the models was evaluated according to the following indices and their respective values: chi-square and degrees of freedom (χ2/gl) ≤ 5.0, the goodness of fit index (GFI) ≥ 0.90, comparative fit index (CFI) ≥ 0.90, Tucker-Lewis index (TLI) ≥ 0.90, Parsimony goodness of fit index (PGFI) ≥ 0.60, Parsimony comparative fit index (PCFI) ≥ 0.6, root mean square error of approximation (RMSEA) ≤ 0.05, P - Root Mean Error of Approximation (PCLOSE) ≥ 0.05 and expected cross-validation index (MECVI); the lower the value, the better.^10^ The relative normed fit index (RNFI) was calculated to assess the quality of the global structural model (Step 2). RNFI > 0.80 is an indicator of good fit and significant trajectories with P < 0.05.^10^

The quality of the local adjustment was identified based on the values of factor loadings (λ > 0.3)^30^ and individual reliability (R² ≥ 0.25).^10^ Modification indices greater than 11 (P < 0.001) were used to refine the models, and the measurement errors that led to considerable improvement in the adjustment of the models were correlated.^10^

### Validity, reliability, and rigor

The instruments used in this study were validated in Brazil. The interviewers collected data from health professionals who underwent training and had qualifications in approaches to ethical research issues. Field supervisors reviewed the interviews to analyze the consistency and completeness of the questionnaire. This study was conducted using a representative sample of older people living in an urban area of a Brazilian municipality.

### Ethical considerations

The project was approved by the Human Research Ethics Committee by Universidade Federal do Triângulo Mineiro, on May 9, 2017 (CAAE:65885617.8.0000.5154). The interviews were conducted after obtaining consent from the participants and the participants signing the Free and Informed Consent Form.

## RESULTS

There was a predominance of female older people (66.9%), those aged 70–80 years (41.4%), those with 1–5 years of education (52.4%) and partners (42.8%), those who lived accompanied by other people (81.1%), and those with an individual monthly income of 1–3 minimum wages (85.8%) (Table 1).

**Table 1:** Frequency distribution of sociodemographic and economic characteristics of older people living in a health microregion, Minas Gerais, Brazil, 2020

Variables	Categories	n	%
Sex			
	Men	317	33.1
	Women	640	66.9
Age (years)			
	60–70	358	37.4
	70–80	396	41.4
	80 or older	203	21.2
Schooling (years)			
	None	171	17.9
	1–5 years	501	52.4
	5 years or more	285	29.7
Marital status			
	Single	63	6.6
	Living with spouse	410	42.8
	Widowed	377	39.4
	Divorced	107	11.2
Housing arrangement			
	Alone	181	18.9
	Accompanied	776	81.1
Monthly individual income (minimum wage)			
	< 1	80	8.4
	1–3	821	85.8
	≥ 3	56	5.8

Table 1 shows the sociodemographic and economic characteristics of older people living the health microregion.

In the QoL assessment, most older people classified it as good (58.7%), followed by not bad/not good (22.3%), very good (13.5%), poor (4.5%), and very poor (1.0%). In the QoL assessment using the WHOQOL-BREF, the highest mean score was for the social relationships (70.12 ± 15.43) domain, and the smallest one was for the physical (64.41 ± 17.15) domain (Table 2).

**Table 2:** Distribution of Quality-of-Life scores of World Health Organization Quality of Life-BREF domains and World Health Organization Quality of Life-OLD facets of older people living in a health microregion, Minas Gerais, Brazil, 2020

Quality of life	Mean	Standard deviation
**WHOQOL-BREF domains**		
Physical	64.41	17.15
Psychological	70.07	14.28
Social relationship	70.12	15.43
Environment	65.88	13.02
**WHOQOL-OLD facets**		
Functioning of the senses	73.82	22.84
Autonomy	69.14	15.58
Past, present, and future activities	69.26	14.44
Social participation	67.20	16.29
Death and dying	75.43	26.73
Intimacy	72.72	19.96

WHOQOL-BREF = World Health Organization Quality of Life-BREF; WHOQOL-OLD = World Health Organization Quality of Life-OLD.

The facet of the WHOQOL-OLD that presented the highest mean score for QoL was death and dying (75.43 ± 26.73), and the lowest score was in the social participation facet (67.29 ± 16.29) (Table 2).

Table 2 shows the QoL scores measured using the WHOQOL-BREF and WHOQOL-OLD for older people living in a healthy microregion.

**Figure 1:**
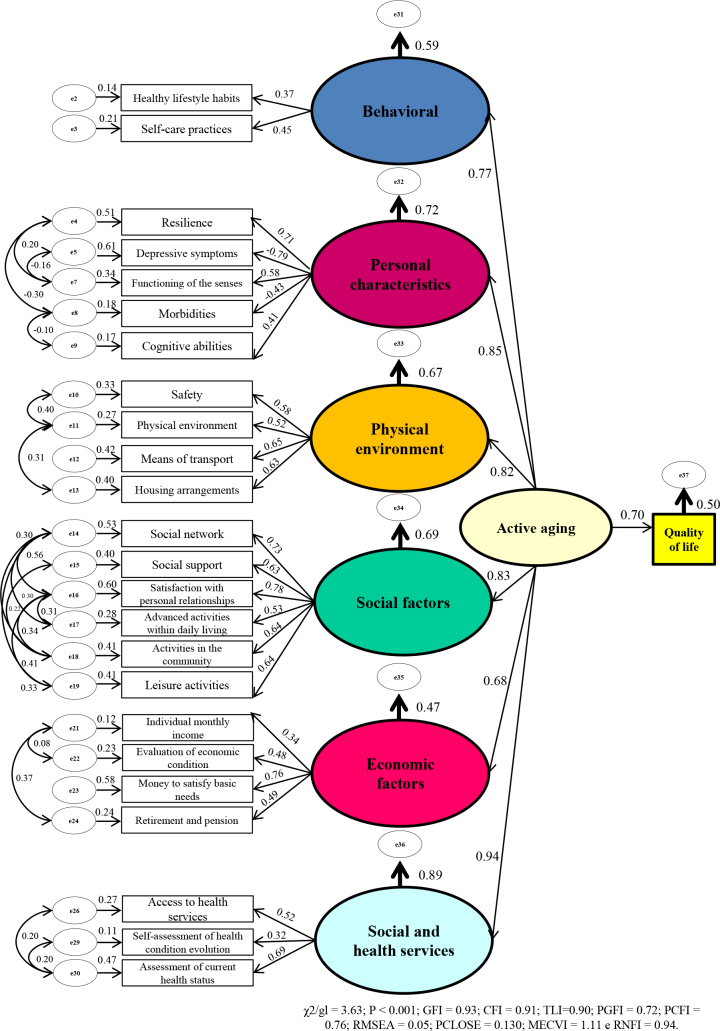
Structural model of the effect of active aging on the self-assessment of the quality of life of older people in the current study.

The structural model, which demonstrates the effect of active aging on the self-assessment of QoL of older people, showed a good quality of adjustment (χ2/gl = 3.63, P < 0.001; GFI = 0.93, CFI = 0.91, TLI = 0.90, PGFI = 0.72, PCFI = 0.76, RMSEA = 0.05, PCLOSE = 0.130, MECVI = 1.11, and RNFI = 0.94) (Figure 1).

QoL = quality of life; GFI = goodness of fit index; CFI = comparative fit index; TLI = Tucker-Lewis index; PCFI = Parsimony comparative fit index; RMSEA = root mean square error of approximation; PCLOSE = P-root mean error of Approximation; MECVI = expected cross-validation index; RNFI = relative normed fit index.

Most items had a standardized factor loading (λ > 0.3) and adequate individual reliability (R² ≥ 0.25) (Table 3).

**Table 3:** Standardized factor loadings and individual reliability of the variables of the structural model of the effect of active aging on the self-assessment of the quality of life of older people living in a health microregion, Minas Gerais, Brazil, 2020

Variables	λ*	R²**	P***
**Active aging and determinants**			
Behavioral	0.77	0.59	< 0.001
Personal	0.85	0.72	< 0.001
Physical environment	0.82	0.67	< 0.001
Social	0.83	0.69	< 0.001
Economic	0.68	0.47	< 0.001
Social and health services	0.94	0.89	< 0.001
**Behavioral determinants**			
Healthy lifestyle habits	0.37	0.14	< 0.001
Self-care practices	0.45	0.21	< 0.001
**Personal determinants**			
Resilience	0.71	0.51	< 0.001
Depressive symptoms	-0.79	0.61	< 0.001
Functioning of the senses	0.58	0.34	< 0.001
Morbidities	-0.43	0.18	< 0.001
Cognitive ability	0.41	0.17	0.002
**Physical environment**			
Safety	0.58	0.33	< 0.001
Physical environment	0.52	0.27	< 0.001
Means of transport	0.65	0.42	< 0.001
Housing arrangement	0.63	0.40	< 0.001
**Social determinants**			
Social network	0.73	0.53	< 0.001
Social support	0.63	0.40	< 0.001
Satisfaction with personal relationships	0.78	0.60	< 0.001
Advanced activities of daily living	0.53	0.28	< 0.001
Activities in the community	0.64	0.41	< 0.001
Leisure activities	0.64	0.41	< 0.001
**Economic determinants**			
Individual monthly income	0.34	0.12	< 0.001
Economic condition evaluation	0.48	0.23	< 0.001
Money to satisfy basic needs	0.76	0.58	< 0.001
Retirement and pension	0.49	0.24	0.009
**Health and social services**			
Health services access	0.53	0.28	< 0.001
Self-assessment of health condition evolution	0.32	0.11	< 0.001
Assessment of the current health status	0.77	0.59	< 0.001
**Self-assessment of quality of life**	0.70	0.50	< 0.001

^*^Factor loading (λ);

^**^Individual reliability (R²);

^***^P < 0.05.

Table 3 shows the standardized factor loadings and individual reliability of the observed variables that comprise the structural model of the effect of active aging on the self-assessment of QoL in older people living in a health microregion.

It was found that acting aging, the second-order factor, explained 50% of the variation in self-assessment of QoL and had a direct and positive effect on this outcome (λ = 0.70; P < 0.001), showing that the more people actively aged, the better their self-assessment of QoL; that is, an increase in one active aging unit implies an increase of 0.70 in the self-assessment of QoL (Figure 1).

## DISCUSSION

In the current study, most older people self-assessed their QoL to be good. It was also found that the highest mean QoL scores were for the domain of social relationships and facet of death and dying, while the lowest was for physical relationships and social participation. Furthermore, a global structural model was proposed to measure the effect of active aging on the self-assessment of QoL in older people living in the urban area of a health microregion in Minas Gerais. Active aging was found to have a direct positive effect on these outcomes.

Data related to the QoL self-assessment, verified in the current study, were obtained from older people living in the city of Uberaba (MG), in which the majority (51.1%) rated their QoL as good.^31^ However, different results were identified among older people from other cities in the same state as those in the research in question, as a higher percentage classified QoL as regular (54%)^32^ and poor (41.3%).^6^ Such differences may be related to ethnic and cultural differences, as these can interfere with subjective measures self-reported by the older people, such as QoL.^33^

In the current study, higher mean scores were observed for the domain of social relationships, similar to studies conducted among older people in the community in Brazil^31,34^ and Greece.^35^ A varying result was shown in a survey in the Netherlands among people aged ≥ 50 years, in which this domain had the lowest QoL scores.^36^ Positive personal relationships associated with an active social life contribute to the prevention of social isolation, reflecting the physical and mental health status of older people and, consequently, their QoL.^37^ In this context, approaches that make it possible to integrate the family and components of the social network into care are resources that should be valued and used as they add to the QoL of older people.^38^

A lower score in the physical domain may indicate a more significant impact on daily activities; dependence on drugs or treatments and work capacity are aspects evaluated in this domain.^3^ Similar data were found in other studies in Brazil,^33,39^ Poland,^8^ and Greece.^35^ This finding highlights the importance of reevaluating the impact of physical health on the QoL of older people, with a view to establishing actions that favor self-care and maintenance of functionality and independence during aging.

The highest average scores in death and dying, verified in the research on screen, align with the findings among older people in Brazilians^34,40^ and differ from studies conducted in the Netherlands, in which this facet was among those with the worst evaluations.^37^ Such data suggest that these individuals are facing, in a good way, concerns and fears related to the end of life, which are items evaluated in this facet.^3^

The lowest scores obtained on the social participation facet corroborate the investigation among older people in the community in Brazil.^40^ It is possible that the lowest scores on the social participation facet in the current study were due to the worse assessment of the physical domain because these QoL items may be associated, as shown in a previous study.^41^ Reducing older people’s social participation is a relevant aspect to consider. Generally, it is multifactorial and includes access to income and socialization difficulties, including physical aspects, which health services must monitor to improve decision-making capacity and life satisfaction.^1,42^

Although the promotion of active aging is considered the main action to face the challenges caused by the demographic aging process and to improve or maintain the QoL of older people,^1,42^ there are critical gaps in the scientific literature regarding structural models that operationalize the concept of active aging in a broad and multidimensional approach. A survey developed with an older Spanish population stands out, in which an active aging model was developed based on the WHO proposal. However, the analyzed outcome variable was satisfaction with life, whose association was direct and positive.^43^

Studies on the association between active aging and QoL have evaluated the association between some variables that make up the determinants of active aging and QoL.^6–9^ A study of community-dwelling older people in Spain using structural equation modeling found that the availability of social support was positively associated with QoL. It was also identified that perception of health and satisfaction with life were the two main variables for understanding QoL, regardless of the age variable, which did not affect the model.^44^ However, the active aging model was tested from a psychosocial perspective, including four latent variables (depression, explicit memory, perceived QoL, and social resources). Furthermore, a selected sample of healthy older people was included, after excluding those with functional dependence, no education, visual and mental problems, cognitive alterations, or other criteria.^44^

Noteworthy, the active aging approach proposed by the WHO includes all people who are aging, including those who are frail, physically disabled, and require care; its main objective is to maintain or improve QoL.^1^ The interest in studying the relationship between active aging and QoL assumes an increasingly essential role in society because of the aging population worldwide.^45^ The way each elderly person faces and experiences the human aging process is also determined by the subjective assessment of their QoL, making it one of the main factors to be considered when analyzing the living conditions of the older population.^4^

Active aging and QoL are considered complementary concepts because QoL is believed to influence how individuals experience the aging process. The ability to remain active during this process is considered a determinant of QoL, whether in maintaining autonomy and independence, which contributes to performing daily tasks, or conducting social activities such as participating in groups and developing voluntary work.^42^

Therefore, given the current state of scientific knowledge, it appears that the findings of this study innovate by showing a direct and positive effect of the global structural model of active aging on the self-assessment of QoL in a representative sample of older people in the community, supporting discussions of a global public health policy to deal with the challenges of the aging population.^1^ Furthermore, the results offer elements to study in the field of gerontology that can provide information that helps in developing and improving its practice, specifically in health care for the older population, to promote active aging and QoL.

This study has a limitation in that it excluded older people with severe cognitive impairment, which may have favored a healthier sample. However, the possibility of selection bias was minimized, as all eligible older individuals were interviewed. Moreover, as a limitation, the non-inclusion of variables related to culture and sex can act as barriers or facilitators in the active aging process and interfere with subjective measures such as self-assessment of QoL. It is suggested that multicenter studies and national surveys should be conducted with representative samples of the older population in different Brazilian states, including the variables of culture and sex, to improve health care for the elderly and their QoL.

## CONCLUSION

In the self-assessment of QoL, most older adults classified their QoL as good. The highest mean scores were for the social relationships domain and the facets of death and dying, whereas the lowest scores were for physical relationships and social participation. Active aging had a direct and positive effect on the self-assessment of QoL, indicating that the more people actively aged, the better their self-assessment of QoL. Therefore, investigations into the determinants of active aging among older people in the community are relevant to establishing follow-up actions in health services. Additionally, primary care nurses had the most contact with older people. Therefore, identifying these aspects helps reflect actions to promote active aging, considering their effect on the QoL of this age group.
